# openBIS: a flexible framework for managing and analyzing complex data in biology research

**DOI:** 10.1186/1471-2105-12-468

**Published:** 2011-12-08

**Authors:** Angela Bauch, Izabela Adamczyk, Piotr Buczek, Franz-Josef Elmer, Kaloyan Enimanev, Pawel Glyzewski, Manuel Kohler, Tomasz Pylak, Andreas Quandt, Chandrasekhar Ramakrishnan, Christian Beisel, Lars Malmström, Ruedi Aebersold, Bernd Rinn

**Affiliations:** 1 Department of Biosystems Science and Engineering, Center for Information Sciences and Databases, Swiss Federal Institute of Technology (ETH) Zurich, Switzerland; 2 Swiss Institute of Bioinformatics (SIB), Switzerland; 3 Department of Biosystems Science and Engineering, Quantitative Genomics Facility, Swiss Federal Institute of Technology (ETH) Zurich, Switzerland; 4 Department of Biology, Institute of Molecular Systems Biology, Swiss Federal Institute of Technology (ETH) Zurich, Switzerland; 5 Faculty of Science, University of Zurich, Switzerland

## Abstract

**Background:**

Modern data generation techniques used in distributed systems biology research projects often create datasets of enormous size and diversity. We argue that in order to overcome the challenge of managing those large quantitative datasets and maximise the biological information extracted from them, a sound information system is required. Ease of integration with data analysis pipelines and other computational tools is a key requirement for it.

**Results:**

We have developed openBIS, an open source software framework for constructing user-friendly, scalable and powerful information systems for data and metadata acquired in biological experiments. openBIS enables users to collect, integrate, share, publish data and to connect to data processing pipelines. This framework can be extended and has been customized for different data types acquired by a range of technologies.

**Conclusions:**

openBIS is currently being used by several SystemsX.ch and EU projects applying mass spectrometric measurements of metabolites and proteins, High Content Screening, or Next Generation Sequencing technologies. The attributes that make it interesting to a large research community involved in systems biology projects include versatility, simplicity in deployment, scalability to very large data, flexibility to handle any biological data type and extensibility to the needs of any research domain.

## Background

Systems biology is a recent approach to life sciences that poses unprecedented computational challenges [1-4]. These challenges are rooted in the way systems biology projects are approached and are specifically the following. Investigations frequently span multiple years and are carried out by multiple cooperating laboratories with complementary, interdisciplinary skills. Large and complex datasets measuring different properties of the system studied are acquired and need to be analyzed and included in theoretical models. The long duration of such projects necessitates the ability to cope with investigators leaving the lab during the project and handing over the data to their successors. Furthermore, data analysts and mathematical modellers also need to get access to all or to a subset of the data for down-stream analysis. The fact that more and more diverse datasets are acquired and analyzed over a longer period of time by a large number of researchers within one project needs to be reflected in the way data are stored, managed, indexed, queried and integrated. Until recently, biologists used file management systems on their personal computers to manage their results, a strategy that is ill-suited for the requirements of data sharing in systems biology research and which does not scale to the data output of modern instruments used for data acquisition.

For a long time, data management in life sciences has been considered a side-aspect of data analysis. Domain-specific analysis procedures are often closely tied in with some sort of data management. Examples of this approach for genomics are MIMAS, MiMiR, GNomEx, and Biological Networks 2.0 [5-8], examples for proteomics include CPAS, PRISM, 2DDB, CPFP, Maspectras 2 and ProHits [9-14]. The advantage of this approach is that it can give researchers a "turn-key solution" if the analysis fits their needs. On the other hand, it may create high migration effort when the data analysis requirements are changing during a project in a way which is not supported by the analysis platform. We argue that this is a common case for long-running projects that use cutting edge technologies.

An alternative approach is to use generic workflow managers and implement the analysis procedures as nodes of the workflow manager. Today, many good solutions for scientific workflow automation are available [15-18]. In order to scale up, workflows can be parallelized to run on grids using middleware systems like P-Grade [19]. The promise of workflow managers is that analysis procedures of heterogeneous provenance can be combined into one workflow and can be run repeatedly and reproducibly, providing the flexibility that the tightly integrated systems are lacking. There are, however, some pitfalls also for this approach. First of all, for each preexisting analysis procedure there is the need to write „glue code" to integrate it as a node into the particular workflow system. Furthermore, as of today no commonly accepted workflow definition language and representation exists, so that workflows created for different workflow managers are mutually incompatible. However, work for creating a common workflow format that is independent of a particular system and that can be supported by many bioinformatics workflow managers is ongoing and expected to become available with OpenMS/TOPP 1.9 [20, personal communications: Kohlbacher O]. Finally, employing a workflow manager without giving consideration to data sources and data sinks used by the workflow usually leads to solutions where data management is reduced to file-based data storage. This will create the usual problems of file-based solutions regarding scalability and the need for tedious manual processes for data provisioning and sharing.

While data analysis methods and algorithms change quickly to follow the latest advances in the field, a data management system needs to provide a stable basis for measurement data and analysis workflows for many years. Data from multiple generations of measurement devices and many versions of analysis pipelines need to be kept available for reference, comparison and integration. During this time, the appropriate data representations (for example a relational data model storing result data) may change as community standards evolve and more sophisticated analysis methods become available. A software ecosystem that is well prepared for such challenges will consist of many software components that fulfill a specific function optimally for a given research project and that can be integrated in the overall pipeline with as little effort as possible, ideally by 'plug-and-play'. To that end, the components need to be easy to integrate with each other and must not try to offer all functionality by themselves but rather provide interfaces to other components which offer the required functionality. Due to the fluid nature of the research process, there is no (and arguably never will be) a formal component model. Thus, the components of such a software ecosystem have to be written with a focus on ease of integration. We argue that a critical component is a domain-specific data management system which acts as a hub for the data being measured and analyzed, providing the data sources and sinks to the various analysis components, as well as being a repository for data sharing. One of its functions is to bridge the gap between incompatible workflow systems used for different analysis phases. To this end, it has to be independent of any particular measurement or analysis pipeline, independent of a particular data representation and able to evolve its data representations and support multiple representations in parallel. To support the necessary integration with analysis pipelines, it needs to be open for extension and integration. Furthermore, it needs to offer strong data provenance tracking capabilities, advanced search functionality and a scalable backbone for data ingestion, provisioning and life-cycle management. An early representative of some of these ideas is the genome database AceDB (A Caenorhabditis elegans database), as it tries to address the flexibility in modelling biological objects [21]. Recent systems that focus on flexible data and metadata ingestion, sharing and querying, as well as integration with analysis systems include SBEAMS [22], B-Fabric [23] and SEEK [24].

Here we report how we addressed the challenges described above by developing openBIS (open Biology Information System). openBIS consists of a core and a framework layer offering services that other software can use via programming interfaces to extend openBIS to specific data types and workflows. The core of openBIS contains a powerful and flexible annotation service that includes support for data provenance tracking, it has a project structure for experiments and data, handles user authentication and authorization as well as data import and export, and supports data format migration and data archiving. Emphasis has been given to provide access to all services of the openBIS framework via application programming interfaces (APIs) that allow integrating openBIS with other software tools for primary and secondary data analysis, visualization and automation. We have also implemented web-based graphical user interfaces based on those APIs. To customize the system for specific technologies such as Next Generation Sequencing, High Content Screening [25], quantitative imaging or mass spectrometric methods used in proteomics and metabolomics research we have adapted openBIS to the specific data structures and annotations, mechanisms for data uploading, visualization and support for domain specific queries. Different laboratories involved in SystemsX.ch, the Swiss initiative in quantitative systems biology, and also other large research consortia are productively using openBIS. We expect that its use will grow as systems biology approaches are becoming more prevalent in the life sciences.

## Implementation

### Guiding principles

openBIS has been designed to be open for extension and integration. It uses extensively the design principle of "loose coupling", which is essential for open software systems. The concept has originally been introduced in behavioral science [26] and since then successfully applied to the design of software systems [27]. The loose coupling approach requires a system to use an interface of another system that requires the least knowledge about it. Loose coupling makes a system agnostic about the operating environment it runs in or components it interacts with and thus allows software developers with different backgrounds and skills to join forces in building larger systems.

One of the consequences of focusing on integration is that in openBIS, all metadata are configurable so that they can match the metadata provided or required by other systems that openBIS is integrated with. The same holds true for the logic of data ingestion procedures. A consequence of following the loose coupling approach is that we focus on programming interfaces for openBIS that can be used from different programming languages and operating systems. For "pull" operations, for example querying for the datasets of an experiment, openBIS provides access to data by either a file system, a web-service or a relational database. As not all programmers are familiar with using web-services, we also wrote some command-line tools which encapsulate one or more web-service calls and can be used as a proxy for the web-service. For "push" operations, for example notifying a workflow system that bulk data have become available on a cluster file system, openBIS uses the concept of message passing. As message channels, we use either files on a file system (which may be accessed remotely via an SSH tunnel), a relational database, or e-mails.

A principle we followed to enable good scalability is the separation of bulk data (which can be raw data or result data) and metadata. As data ingestion, provisioning and querying on high-throughput and high-content data can involve expensive operations in terms of CPU usage, I/O and network bandwidth, these operations should not be performed on the same system that maintains and provides access to the metadata which deals with comparably small amounts of data and thus can easily scale up to many experiments and samples. In openBIS, we introduced the *Application Server *(for a glossary, see Additional file 1), short *AS*, for dealing with metadata including data provenance data and one or more *Data Store Servers*, short *DSS*, that deal with bulk data. All communication of the AS with a DSS is asynchronous.

A principle openBIS adheres to in order to provide good traceability of results is that of immutable datasets: a dataset, once created, has a unique identifier and cannot be changed anymore. When data are derived from one or more datasets, a new child dataset of the original dataset(s) is created rather than changing the original dataset(s). This way, a dataset has a well-defined meaning, for example when referring to it as an input of a data workflow.

Furthermore openBIS is independent of a particular data representation. Whatever representation or format a data measurement or analysis pipeline delivers can be stored in openBIS without the need of changing or transforming it. If a second representation is required or an index of the data needs to be built for later querying, this can be done when running the data ingestion procedure and provided as a separate representation. We encourage users to create a new dataset for each representation and put different representations of the same data into one "container dataset", which acts as a proxy to all representations. To make a container dataset transparent to the user, openBIS will show its file system view by fusing the files of all contained datasets. New representations can be added at a later time by transforming the old representation, creating a new dataset from it and adding that to the container dataset, a process which can be run as a background thread of a DSS.

### openBIS overview

**Figure 1:**
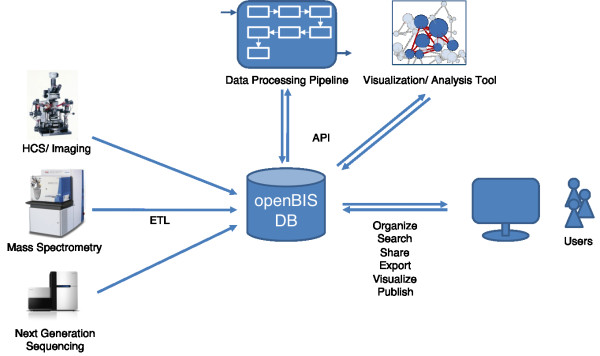
**openBIS overview**. openBIS is a data and information system supporting users at every level from data collection/organization to publication. It supports research workflows from one or several different sources such as microscopes, mass spectrometers or DNA sequencers. Via APIs the system can be extended to integrate tools for data specific analysis and visualization.

The main goal of openBIS is to support biological research data workflows in the realm of systems biology from the source, for example the microscope, the mass spectrometer or the sequencer, to data publication. This is accomplished by creating an information system which extracts data and metadata from the measurement instruments, integrates with data processing pipelines and visualization and analysis tools, and allows users to interact with the system in multiple ways (Figure 1). For the sake of brevity, we will use the term *data processing *whenever we refer to primary data analysis and *data analysis *when we refer to secondary analysis. Data from the different measurement platforms are uploaded into the system using *dropboxes*. A dropbox corresponds to a directory on the file system that is monitored for incoming files and directories. Copying data in one of the configured dropboxes triggers the ETL (Extract, Transform, Load) process which extracts metadata, creates datasets annotated with metadata in the database and adds datasets to the data store. Integration with other computational tools is achieved via APIs. Users can access openBIS to organize, search, share or publish data and metadata. Data can be exported to Excel. Bulk export is provided by a web interface or command line tool. Overall, openBIS is a flexible system allowing users to access raw and processed data from different downstream applications and track data provenance, independent of the instrument or software vendor, independent of geographical location of the instrument for a longer period of time. Due to its architecture openBIS is very scalable, i.e. it can store and make available large amounts of data and is configured for distributed storage.

### Logical structure of the data repository

**Figure 2:**
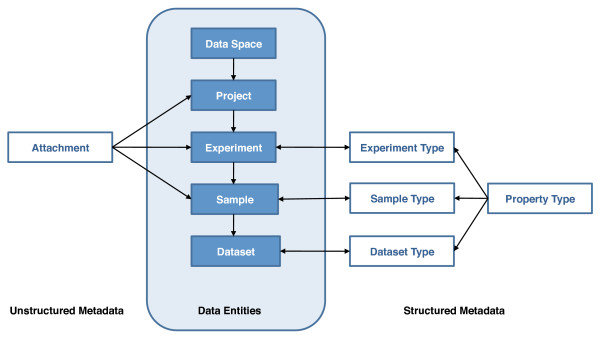
**Data organization and metadata**. Data are organized using entities and relations that are familiar to scientists. To organize metadata, the concepts of experiment, sample and dataset types have been introduced. Structured metadata will be assigned at the respective levels by using Property Types. Property Types are unique and can be reused and allow researchers to provide custom properties (or annotations) to experiments, samples and datasets.

To organize the stored data in a logical and transparent manner and to manage access privileges we created a hierarchical structure of the data. This was accomplished by using the entities i) *Data Space*, ii) *Project*, iii) *Experiment*, iv) *Sample *and v) *Dataset *(Figure 2). Permission rules are applied at the highest level, the data space. These rules determine what a user is allowed to see and which operations he is able to perform. A data space contains projects that group one or more related experiments. An experiment is an empirical approach to acquiring data. The experiment in turn typically contains at least one sample, the object being measured or observed in an experiment. A sample can have one or more datasets associated with it, where a dataset is a set of files containing the values of the actually measured or derived data. For example, the same microtiter plate (sample) being read twice by a microscope will result in two different datasets which both can be associated with a single sample. Such a hierarchical structure is a prerequisite for organizing larger collections of experimental datasets efficiently. For example, both raw data and processed data can be stored as individual datasets which in turn are linked to each other and to a sample or an experiment. The hierarchical data structure further is capable of establishing parent-child relationships between samples and between datasets.

### Metadata

To describe the structure and meaning of data with all its contents in context, and to facilitate finding and using acquired data, metadata are stored and indexed along with raw and analyzed data. Metadata in openBIS can be either structured or unstructured and can be provided and searched for in a flexible way. We support unstructured metadata through file attachments at the level of the project, experiment or sample, very much like an e-mail attachment. Alternatively, structured metadata allow researchers to provide custom properties (or annotations) to experiments, samples and datasets that are defined as a set of *Property Types *which are stored in the database in a consistent and transparent manner (Figure 2). A Property Type can be regarded as a field definition with a name, label, description and value where the value is constrained to be a known data type such as an integer, a floating point number, a date, a boolean value, a free-text field, a hyperlink, a term of a previously defined Controlled Vocabulary or, for semi-structured metadata, an XML structure. Property Types are subsequently assigned at the level of the experiment, the sample or the dataset by linking them with a given *Experiment Type*, *Sample Type *or *Dataset Type*. Properties can be dynamic, i.e. they can be evaluated using a script based on other metadata in the system. Due to this flexible annotation service, openBIS provides a generic mechanism for establishing metadata models that are often specific to an experimental venture. To effectively handle research specific annotations or attributes, openBIS uses an entity-attribute-value (EAV) data model [28].

### Data repository

In order to efficiently store and retrieve the large amounts of data associated with systems biology studies and to cope with many different measurement technologies, openBIS employs a hybrid *data repository*, featuring a *relational database management system *(RDBMS) [29] for index information, metadata and selected results, and a flat-file *data store *for bulk data. By default, raw data and result data are stored in the data store which is a managed flat-file store consisting of one file system (*flat storage mode*), or multiple file systems (*segmented storage mode*). Experiment-related metadata are always stored in the RDBMS, file metadata may be stored there optionally. The hybrid data repository combines flexibility and scalability. The system is flexible because it stores the metadata model reflectively in the database, i.e. the model *is *data itself. Therefore, the metadata model can be chosen for each individual use case whether data are best represented in the original data files or, for time-critical queries, in an optimized file format or the RDBMS. The system is scalable because i) the core database (which resides on fast storage) is kept at a reasonably small size, ii) the queries which regularly deliver very large result lists are optimized, and iii) there is support for segmented and distributed storage of bulk data.

### openBIS deployment model

**Figure 3:**
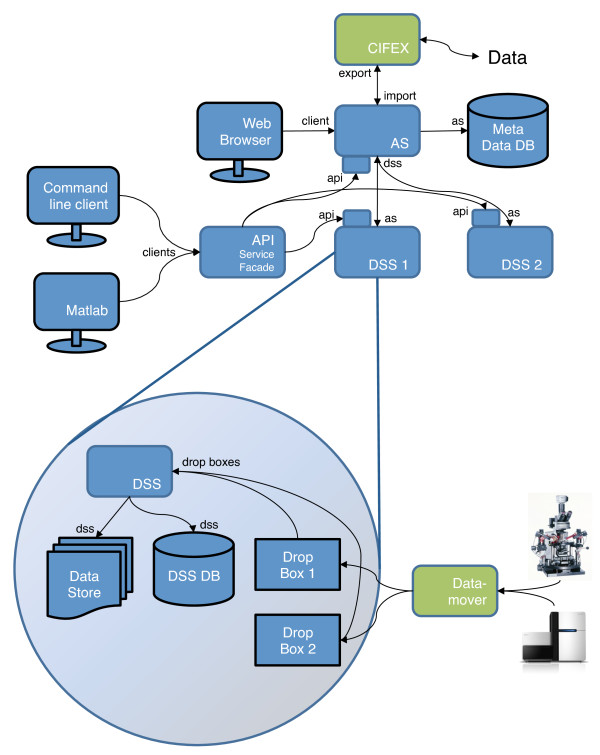
**openBIS deployment model**. The overview is shown in the upper half, a more detailed description of the DSS is shown in the lower half. Companion Servers are shown in green. Arrows indicate how the components interact with each other.

The basic openBIS deployment consists of two servers, the Application Server (AS) and the Data Store Server (DSS) (Figure 3). Generally speaking, the AS manages the metadata and links to the data while the DSS manages the data itself. To this end, the AS sets up and uses an RDBMS to persist users, authorization information, entities like data spaces and samples and their metadata, as well as index information about all datasets. The DSS manages the datasets in the data store, which is not writable by other parts of the system. Different types of clients like e.g. a web browser, a graphical Matlab client, or a command-line client can access openBIS through the AS and DSS.

One AS can manage multiple DSSes. This is a feature that is important e.g. if the data are acquired in multiple, distributed laboratories with a local DSS at each site. A DSS, by design, is not just an 'internet-ready file server'. It can run custom queries on the data it stores, it may (and in many cases does) employ its own RDBMS for result data that it will populate automatically on data upload. Furthermore the system can be extended to use additional *Companion Servers *for data transport, such as the *Datamover *or *CIFEX *for data import and export (described below).

### Client

In order to interact with the system, users need to use a client. Out of the box, openBIS provides a graphical web application, a set of command-line tools, which communicate with openBIS over the network, and a few KNIME [16] nodes. In many cases, users will use a client that is specific to their domain and use case. To support use of the openBIS backend from such clients, we provide APIs that are accessible over a TCP/IP network. Clients come in many forms and flavors and the writers of the client software are inventive in how they integrate. The methods we see being used beside the APIs include setting a link to a view of the openBIS web application (it offers permlinks for that purpose), calling an openBIS command-line tool, writing to a dropbox directory and let the dropbox script write response files, or accessing custom relational databases that are populated by a dropbox script.

### Application server

The AS is the central point of access for clients who wish to interact with the system. It stores metadata about samples, experiments, and datasets in an RDBMS. The actual data is stored and managed by the DSS; the AS mediates access to one or several DSSs.

The AS itself can be decomposed into several layers: the presentation layer, the domain layer, and the data access layer (Additional file 2). The presentation layer serves the HTML and JavaScript for the web client. The domain layer provides services for clients wishing to interact with the business objects (entities such as samples, experiments, etc.). These services are not only used by the web client, but by command-line programs and the DSS as well. Example services include queries such as samples matching particular criteria, and registering entities in the database. The data access layer is the interface between the AS and the database; it is private to the AS and not accessible from the outside.

### Data store server

The data that comprise a dataset, be it images, spectra, analysis results or features, are managed by and accessed through the DSS. These data are stored in the data store. The DSS is responsible for querying, reporting on and creating visualizations of datasets.

In flat storage mode, the data store will consist of one share which in general will be served by a file server (e.g a NAS head). In segmented storage mode, one data store has multiple data shares, where different file servers could serve each share. The DSS manages the fill state of the shares in a background process.

The DSS consists of a presentation layer, domain layer, and a data access layer (Additional file 2). The presentation layer is responsible for displaying data. In the most basic cases, this may just be a matter of providing access to an image via a URL for use in an HTML "img" tag. Slightly more involved, but still quite simple, would be a case of rendering a tab-separated-value file in the form of an HTML table. More complex cases require a deeper understanding of the data. Examples of more complex presentation logic in the DSS include generation of data visualizations, such as heat maps, and compositing images representing multichannel data. A part of the DSS presentation layer is the Data Set Uploader, a tool for web-based batch upload of data sets. To avoid the web browser's size limitation of 2 GB for uploaded files, this tool is implemented in Java and executed via Java WebStart.

The domain layer of the DSS is responsible for providing access to the data and understanding their format and semantics. Also in the domain layer are the ETL threads which are responsible for registering new datasets when they arrive. In the typical use of the DSS, an ETL thread watches a folder for activity and registers the data when a new file or folder appears. The data access layer of the DSS consists in the generic case of a filesystem abstraction. This abstraction allows the DSS to use file metadata from an RDBMS to speed up search and list operations on slow storage, transparent access to container files (an HDF5 container shows up as a directory) and fusing the directory listings from multiple datasets being in the same dataset container. This layer may furthermore provide access to additional data sources like bespoke relational databases.

### CIFEX

Beside openBIS, we have developed other applications that can be used in concert with openBIS: CIFEX and Datamover. CIFEX, the CISD File EXchanger, is a web-based application for exchanging and transferring large files. As the web browser limits uploads to 2 GB, CIFEX has a Java WebStart-based GUI tool to overcome this limitation. It supports resuming up- and downloads and performs checksumming to ensure file integrity. We have integrated CIFEX with openBIS to enable downloads of large or huge datasets that are available in openBIS. If a user has direct access to the file system of the data store, CIFEX is not required, but many users do not have that access and furthermore CIFEX can be used to share a selected subset of data when a user to share with does not have an openBIS account. For this use case, e-mailing datasets as attachments is often not an option anymore as they can be multiple gigabytes in size. For these use cases, CIFEX functions as a conduit for transferring data to and from openBIS (Figure 3).

We run an instance of CIFEX for users of ETH Zurich. The CIFEX system is open source and available for download [30].

### Datamover

In order to reliably transfer data from one location to another the Datamover program was developed. It can use either a locally mounted file share, an SSH tunnel or an rsync server for the actual transfer. One example is transferring data produced by a measurement device attached to a dedicated measuring computer (Figure 3). The files produced are often very large, the storage space available on the measuring computer is limited, and network access to the storage repository may not be entirely reliable at all times. Datamover detects new files on the measuring computer and transfers them to another location such as the data center or compute cluster. It thus robustly and automatically handles the potential pitfalls that may occur in the data transfer process such as network interruptions or storage devices reaching their capacity limit.

### Authentication and authorization

Each user must login to gain access to openBIS. openBIS features a plugin-based system for authenticating users. Authentication plugins are available for LDAP v3 (using e.g. Microsoft Active Directory as the LDAP server), Crowd, an identity management system written by Atlassian [31], and a simple file-based approach (analogous to the passwd file of Unix systems). Within the application access permissions are defined by assigning *Roles *to users or groups of users. A role determines (authorizes) what a user or group of users is allowed to see and what operations a user can perform. A user having administrative permissions can grant access rights to other users or user groups to facilitate sharing of data. As a result of the established authentication and authorization service access to secured information can be restricted to particular users based on the role each particular authenticated user has in the system. Thus, unauthorized users are prevented from gaining access to secured resources.

### Usage and applications of openBIS

Users can interact with openBIS in several ways. The software framework provides a set of commonly required core functionalities, such as the management of data and metadata, the search capability for and the sharing of data, the import and export of data, and the interfacing with the system per se. These core functionalities are stable and part of the openBIS download package and thus can be used 'out-of-the-box' by biologists. Integration of openBIS as a new system in a specific research workflow very often is achieved by implementing additional custom functionalities, such as visualization, publication and bespoke query operations of the data. Implementing such custom functionalities requires a trained software developer and usually a tight interaction between the scientist and the software developer. For an overview of openBIS functionalities see Additional file 3.

### Data and metadata management

To organize data in a logical and safe way researchers apply a hierarchical data structure using the openBIS entities (Figure 2). For each research group a data space is created to which permission and visibility rules apply that are defined by the access privileges. Access permissions can be defined by assigning for example an observer (read-only access) or user role (additional permissions to add and edit entities) to individual users or user groups to keep all data restricted to all members of a project. Following publication, researchers can easily change permission settings to make their data and metadata publicly available. Within each data space each research group can organize its projects. A project typically contains the experiments which contain all the datasets and metadata files created from the individual samples run by a particular measurement instrument. To provide custom annotations to datasets, samples or experiments, sets of property types are defined which in turn are grouped and assigned at the level of the experiment, sample or dataset type, respectively. Using these capabilities, custom metadata sample submission and update forms can be created.

### Data search, export and sharing

To share data in collaborative projects the dataset search functionality in combination with the application of table filters and the bulk export functionality using CIFEX can be utilized. Datasets are searched based on dataset code, dataset type (e.g. *raw data *or *protein results*), file type (e.g. *mzXML *or *protXML*), or any other metadata stored for the dataset, sample or experiment (e.g. *conversion software *of *file name*). Applying a filter on it allows the user to find the relevant entries. Standard filters are applied to filter the content of a table column. Numerical filters allow numerical comparison operations. Finally selected data can be conveniently exported through the spreadsheet export functionality or downloaded by the export data functionality. This will redirect the user to the file exchange system CIFEX. This way openBIS can be used to share data, for example, for down-stream analysis by mathematical modellers.

### Data import

For different usage modalities data are uploaded to an openBIS DSS using one of three methods: a dropbox, the web-based upload or a custom application using a remote API (Figure 3).

Dropboxes are directories located either on a local file system or on a file server that are monitored for write activity by the DSS. If the DSS finds new data in a dropbox, it will execute an 'Extract Transform Load' (ETL) routine configured for this dropbox. The ETL routine will perform all steps that are necessary to make the new dataset known to openBIS, link it with the appropriate entities (e.g. samples), extract metadata required for searching and transform it to a pre-defined format that may be needed to facilitate querying or integration with other tools (for example computational tools). The ETL routine has many degrees of freedom on what to do with the data arriving and is designed as a set of configurable plugins. However, we recommend that the ETL process also stores the original data without modification in the data store if storage space permits.

Using the Datamover as a companion server, dropboxes can be extended to bridge the Internet, possibly crossing institutional boundaries. We found dropboxes to be a very efficient 'loose coupling' interface between systems that need to exchange bulk data. While designed and optimized for unattended operation, some laboratories prefer dropboxes even for users uploading data manually.

The web-based upload tool can be triggered from the web client. For the actual data upload step, it will redirect the user either to the Data Set Uploader program or to a CIFEX server.

The remote API is a set of Java classes that provides a streaming-based programming model to upload any set of files which constitute a dataset. Compared to dropboxes, it allows for a tighter integration of the application using it, can provide immediate feedback (as it runs synchronously) and supports upload over any WAN that allows for HTTPS traffic without additional network configuration. As the use of Java as a programming environment can be limiting in some cases, openBIS also contains a command line tool for data upload that uses the remote API internally. This tool has been used to integrate the openBIS upload with e.g. LabView- or script-based data providers.

### Interfacing: web interface, command-line tools, APIs

To ease the interaction with openBIS, different interfaces optimized for particular use cases have been implemented: a web application, command-line tools, and Java language-based remote APIs (Table 1). The web application runs on all modern web browsers, making it accessible to users of the popular desktop operating systems (Windows, MacOS X, and GNU/Linux) without prior software installation. It is designed for interactive use (using AJAX to avoid page reloads) and provides amenities such as automatic defaulting of values and immediate validation of user input.

**Table 1:** Interfaces for Interacting with openBIS

Interface	Execution Environment	Target Audience
Web GUI	Web Browser	General Users
Command Line	Command Shell	Power Users/Developers
Java API	Java Virtual Machine	Developers

The command-line tools are interfaces to openBIS designed for interactive use by expert users and automated use in scripting environments. They lack the data input conveniences of the graphical interface, but in exchange support precise and concise data input. The rigid and terse interaction style of these tools make them well suited for employment in the integration of openBIS into automated processing pipelines or into custom-built user interfaces, particularly in cases where the APIs cannot be used.

The openBIS APIs come in two flavors: a Java service facade and a JSON-RPC web service. The Java service facade provide programmers with classes and methods for integrating openBIS into their applications. By using these APIs, a laboratory or research group can seamlessly embed openBIS into their own, custom-developed, applications, such as workflow or visualization tools. The APIs, though implemented in Java, are not limited to use in Java-based applications. Many modern data processing environments, such as Matlab and R, also provide functionality for utilizing Java APIs. In fact, we offer an API variant of the HCS-API for Matlab, designed to support the idioms of the Matlab language. The JSON-RPC web service, while not as high-level as the Java service facade, is usable in all computing environments that support web standards. In particular it enables usage of the openBIS backend from any web application. We found that small web apps that specifically cover one use case are often more appealing to casual users than the built-in openBIS web application that combines all functionality but may be perceived as complex.

### Data visualization and publication

To visualize data it is common to integrate specific visualization tools with the openBIS framework. A simple way is the implementation of a reporting plugin for the display of informative html files like, e.g. those generated by an Illumina HiSeq 2000 sequencer's software. More sophisticated visualization tools are for example the Plate Viewer displaying the layout of a multititer plate or the visualization of feature vectors as graphs, or the Protein Viewer (Figure 8A) displaying protein identifications and quantifications as implemented for customizations for HCS and proteomics, respectively. Integration with an already existing viewer such as an image intensity-rescaling tool are implemented using an API. Regarding the publication of data, simplified views of the whole system can be easily implemented by reconfiguring some parts of the openBIS graphical web client. From the different views listed it is evident that based on the openBIS framework almost any kind of viewer can be implemented for any data type of interest. It is also not uncommon for a web app to use both the JSON-RPC web service interface and custom openBIS views where applicable.

### Data query

In openBIS it is straightforward to amend a database with result data and metadata when a new dataset is added, and thus build up specialized result databases. Such result databases can be used along with the core database to create sophisticated queries (using the Structured Query Language) that go far beyond the metadata search functionality described above, store them in the openBIS core database and make them available to biologists who would not be able to formulate such queries themselves. Queries have names and descriptions, can be parameterized and the query results are shown in a tabular web view and available to users for interactive searching and exporting to Excel.

## Results

### Applications

openBIS is a framework for information systems that can be adapted for individual use cases. This is done by developing extensions to the base system and by configuring the system including the extensions to the operating environment.

Developing a new extension for openBIS requires close communication between the researchers using the system and the software developers. We find that an iterative process between users and developers is required to get a refined solution. The users describe what they need to do with the system and give their input on how they would like it to work, the developers work out a design that they can implement. Focusing on the most important aspects of an extension first and quickly providing an implementation allows the users testing their workflow with the real software in order to refine their requirements.

To illustrate how extensions are developed, two rather different examples of established applications are presented. Our setup of openBIS for a Next Generation Sequencing core facility represents a straightforward measurement workflow while the setup for proteomics data is part of a complex data processing workflow employed in a large distributed research project. The Next Generation Sequencing extension took about 3 person months to develop, the Proteomics extension in comparison about 9 person months.

### Next generation sequencing

The openBIS framework was extended for a core facility to organize the daily workflow and to manage and distribute Next Generation Sequencing data and metadata files. The current instance hosts datasets from more than 1000 sequencing samples from over 40 different research groups with about 200 users.

**Figure 4:**
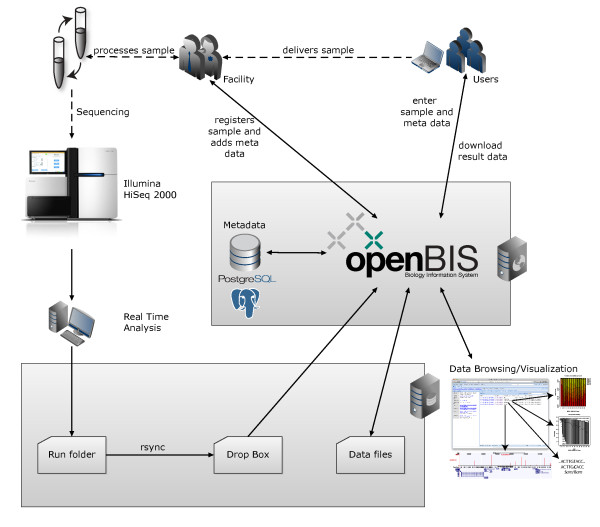
**Work and data flow of openBIS for Next Generation Sequencing.** Integration of openBIS in the workflow of a Next Generation Sequencing core facility. Users can access openBIS through the openBIS webclient to enter new samples, track sample state and to download result data. Facility personnel registers samples after delivery and use openBIS to organize the workflow and to add processing quality control and metadata to the corresponding samples. Sequencing data undergoes real time analysis and the processed data is stored and connected to the samples metadata. Users receive a notification once the sequence data is ready for download.

We customized the openBIS framework to provide the core facility with a sample submission and tracking system (Figure 4). Users can enter the sample metadata within the respective incoming data space of the corresponding research group. Once the biological sample arrives at the core facility a facility member moves it from the incoming data space to the effective data space, where the metadata are only editable by the core facility personnel. The current metadata sample submission form and the sequencing services are configured for Illumina sequencing. They can easily be adjusted to alternative sequencing platforms by defining new sets of property types or by using metadata annotation systems provided by the respective instruments. A tracking system provides an automated e-mail functionality establishing a communication link between the core facility and the researchers indicating the status of key steps in the experimental process, such as the completion of DNA library processing and the generation of quality control and sequencing datasets.

This use case of openBIS is similar to GNomEx, a Genomic Experiment Data Repository and Analysis Project Center [7].

The Next Generation Sequencing data and metadata are organized hierarchically in openBIS. For each research group a data space is created where permission and visibility rules apply. Within the individual data space each research group can organize its projects. A project typically contains the experiments, which contains data and metadata files derived from one sample measured in one flow lane.

Three types of samples have been created for modelling the sequencing setup: First, the 'Illumina Flow Cell' sample type encompassing all eight flow cell lanes containing all raw data as they are provided by the sequencer. The access to this sample type is restricted so that only core facility members can view and download data of a complete flow cell. Through the implementation of a reporting plugin informative html files generated by the Illumina pipeline software like run summary and status reports, demultiplexing statistics, error curves and intensity plots can be displayed. Additionally, it is possible to compare several runs concerning different parameters such as absolute numbers of reads and sequence yield, which are based on the Illumina summary report.

Second, the 'Illumina Flow Lane' sample type containing the sequence data files (FASTQ or SRF [32]) and the metadata as tsv or SOFT files. By request from the customer additional files such as wig/bigWig, bed/bigBed, sam/bam or quality control files are provided. Third, the 'Illumina Sequencing' sample type containing the metadata for the original and processed biological sample as they are entered into the system. The multiplexing of barcoded samples in one flow lane can be covered in openBIS because the relation between the Illumina Flow Lane and the Illumina Sequencing sample types is n:m. This means several Illumina Sequencing sample types are connected to one Illumina Flow Lane (n:1). But also the opposite case (1:m) occurs when an Illumina Sequencing sample is re-sequenced. Therefore, the metadata has not to be re-entered. A facility member simply adds the connection between the two samples.

To give researchers access to their sequence data and metadata they have two options. Either they click on a link contained in the tracking e-mail or they log in to the openBIS application. Within the sample browser one can look through samples according to sample type or data space. Simple text searches as well as advanced, criteria-based regular expressions searches can be performed on experiments, projects and samples.

To export data conveniently the spreadsheet export functionality was implemented. For example, a group leader wishes to get a list of all control samples, which have been sequenced in his laboratory. Within the sample browser filters can be applied for any data column, subsequently data can be exported to Excel. Finally bulk export of data or metadata files can be performed from all dataset tables (e.g. via the advanced dataset search option), which is supported by CIFEX.

As an example of the Next Generation Sequencing extension we have uploaded the datasets of a functional genomics experiment to data space QUANTITATIVE_GENOMICS_FACILITY of the openBIS demo instance [33]. The experiment is comprised of ChIP-seq data (chromatin immunoprecipitation followed by NGS analysis), which led to the conclusion that Polycomb group (PcG) proteins preferentially target promoter regions [34]. PcG proteins constitute a major chromatin based gene silencing system that is required for stable and heritable maintenance of repressed gene expression states [35].

**Figure 5:**
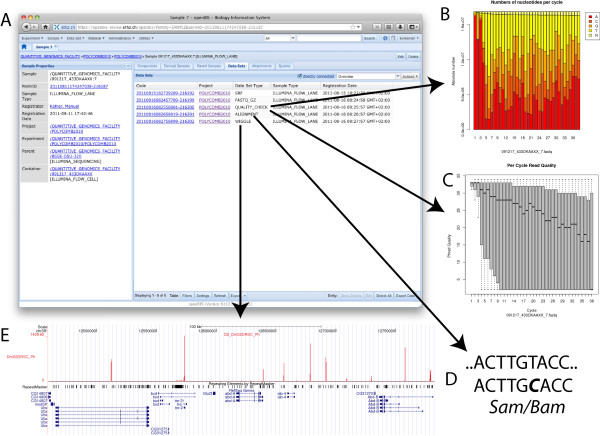
**Data Browsing and Visualization.** A Data Set Browsing. Different datasets generated from the original sequencing data can be browsed. Here five different dataset types are attached to one sequencing sample. B Nucleotide distribution plot. Barplot showing the distribution of nucleotides per sequencing cycle. C Quality plot. Histogram showing the Phred quality scores distribution per sequencing cycle. D Data. Aligned Data stored in SAM/BAM file format. E For visualization of the ChIP-seq data the Wig file can be exported to the UCSC Genome Browser [8]. The screenshot shows a typical binding profile of the PcG protein Polyhomeotic (Ph) in the homeotic cluster BX-C.

The sequencing data of one flow lane sample can consist of several datasets (Figure 5). These datasets are not fixed and can be defined by the openBIS instance admin. In Figure 5A five different datasets are shown: SRF, FASTQ_GZ, QUALITY_CHECK, ALIGNMENT and WIGGLE. Each of these datasets holds files and folder. By clicking on the datasets one can download the data or in case of pdfs or images like in Figure 5B and 5C the files are shown within a frame, depending on the used browser. Both images mentioned before are descriptive for the produced sequencing data and can give first hints on problems about the run. Figure 5E is not viewable in openBIS directly. The created Wig file needs to be uploaded to the UCSC Genome Browser [36] and viewed therein. Although it would be extremely efficient to use a bigWig file and simply reference from the UCSC Genome Browser to this file stored in openBIS, offering a secure access via https, username and password is currently not supported by the UCSC Genome Browser.

### Proteomics data

**Figure 6:**
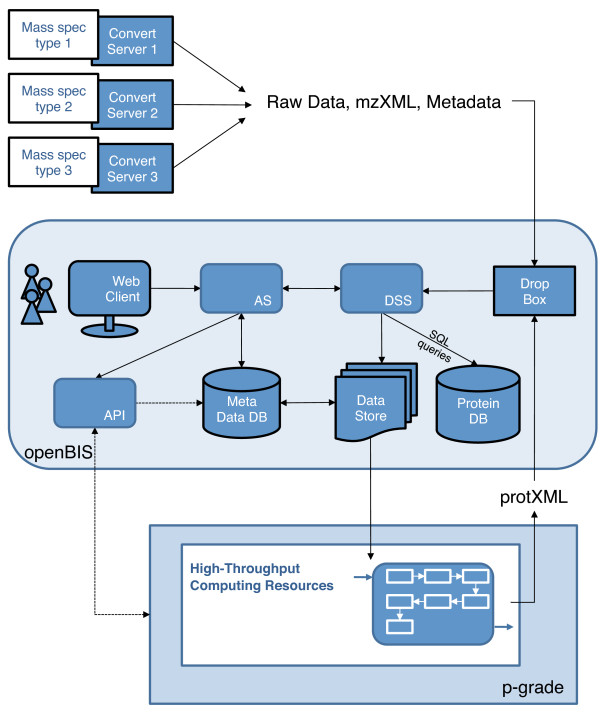
**Integration of openBIS in a typical proteomics workflow. **Raw mass spectrometry data and metadata are uploaded into openBIS using dropboxes (via the web interface, file system or remote server). The user can access openBIS through the openBIS webclient to organize, search and share data and metadata. A web portal (P-Grade) is integrated via a set of dedicated portlets making use of the openBIS API to support the building and execution of proteomics computational applications using grids and other computing resources and to hide the complexity of the underlying infrastructure. Processed data such as protXML (stores protein identifications inferred from input lists of peptides as well as quantification data) resulting from processing pipelines are finally imported into openBIS. Quantification data are added to protXML files by using the parameter feature of the protXML schema.

openBIS for proteomics was developed to manage measured and derived (protein identification as well as protein quantification) data generated in the context of a large interdisciplinary systems biology project of SystemsX.ch. Typically data are produced from different samples measured by several different types of mass spectrometers and need to be consistently processed and transparently stored. openBIS serves as the central hub in this project (Figure 6). Tandem mass spectrometry (MS/MS) data from multiple instruments are converted into an open standard data format (mzXML) [37] and subsequently imported into openBIS. Metadata from the instruments and the conversion are automatically captured and stored in openBIS. The system has currently datasets from more than 100000 injection samples and is used by more than 100 users.

Processing of the data is executed by P-Grade [19]. P-Grade is extensively using the openBIS API, datamovers and dropboxes to facilitate data selection, raw data export and derived data import, respectively. The result of the protein identification and quantification workflow is automatically uploaded into openBIS. Metadata associated with the search are routinely captured and stored in association with the derived datasets.

We demonstrate the utility of the openBIS proteomics extension by using a recently completed study [38]. Streptococcus pyogenes (S. pyogenes) were grown under various conditions gaining insights in various proteins having a role in infection. The bacteria were incubated with albumin, fibrinogen, immunoglobulin G and plasma. MS/MS analysis of these differentially treated samples were compared using openBIS. The data of the corresponding experiment have been uploaded to data space PROTEOMICS of the openBIS demo instance [33].

**Figure 7:**
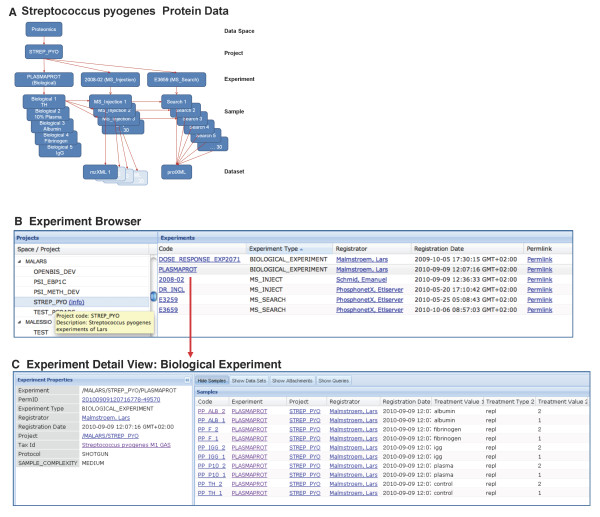
**S. pyogenes data: from overview to detail. **A Data organization. The S. pyogenes project contains three types of experiments, the biological experiment (PLASMAPROT) containing five S. pyogenes samples treated differentially, the MS Injection experiment (2008-02) containing three MS injection samples for every biological sample and the MS search experiment (E3659) containing one MS search sample for every MS injection sample. Every MS injection sample results in one mzXML dataset and all search samples result in one protXML dataset which is generated from all mzXML files used in the identification run. B Experiment Browser. Data spaces and projects are represented by a tree view, project descriptions appear in tool tips. From experiment names one can link to the detailed view. C Experiment Detail View. In the left panel experiment properties (metadata) are listed. In the right panel samples associated with the experiment are listed. Property types (e.g.Treatment Value) and property type values (e.g. albumin) assigned to the sample type are listed for every sample.

To organize the data in a logical and transparent way the hierarchical structure using the openBIS entities was applied (Figure 7A). Within the Proteomics data space the S. pyogenes project is created which in turn contains three experiment types (Figure 7B). Permission settings are configured at the data space level such that normal users have observer rights but no editing rights. The biological experiment (PLASMAPROT) includes the biological samples in duplicates derived from S. pyogenes cultivated under different growth conditions (growth medium alone or supplemented with plasma, albumin, fibrinogen or immunoglobulin G) (Figure 7C). The MS injection experiment (2008-02) includes the MS injection samples and the respective datasets (one mzXML and raw data file for each MS injection sample). The MS search experiment (E3659) includes the MS search samples and one dataset, a protXML file. In addition, metadata about the search are captured as attributes. In this example, as described above, the protXML file contains both protein identification and quantification data.

**Figure 8:**
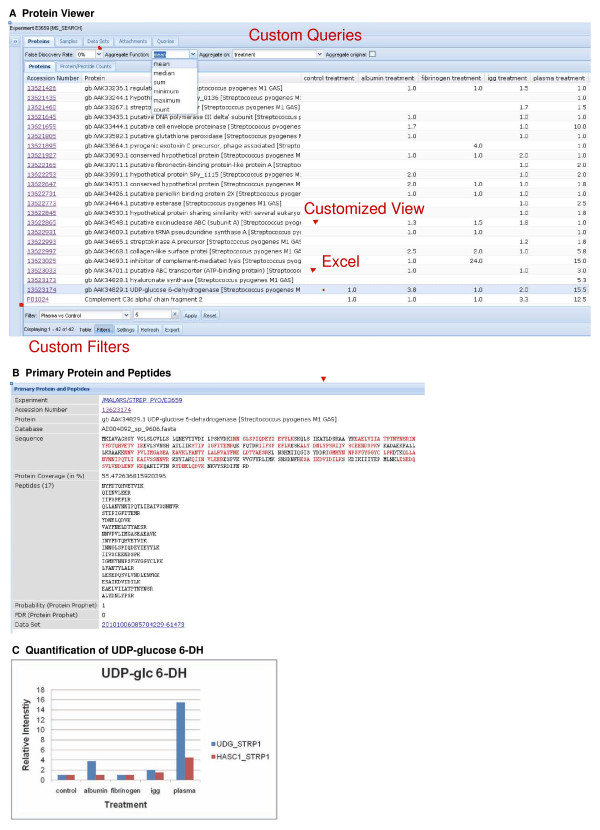
**Drill-in on proteomics data. **A Protein Viewer. Protein identifications and quantifications are listed in a tabular view. A threshold for five pre-computed false discovery rates can be set. Expression based custom filters (bottom) and custom queries based on calculated columns (top) allow the user to further browse the data. Data can be downloaded using the spreadsheet (Excel) export functionality. B Protein and peptides amino acid sequences. Clicking on the accession number in the protein viewer one is redirected to the Primary Protein and Peptides view. From there one can link to external database entries such as UniProt Knowledgebase or GenBank. C Quantification of UDP-glucose associated enzymes by MS analysis. Relative mean protein abundance values of UDP-glucose 6-dehydrogenase (UDG_STRP1) and UDP-glucose pyrophosphorylase (HASC1_STRP1) are plotted against five different treatment conditions.

To visualize both identification and quantification data, a customized Protein Viewer has been implemented (Figure 8A). Researchers can individually customize the view of this table by defining personal table settings to show or to hide columns or to set the sequence of the columns. In Figure 8A one can see that incubating S. pyogenes with 10% plasma induces the expression of UDP-glucose 6-dehydrogenase (Figure 8B). This protein is required for the synthesis of the hyaluronic acid capsule of S. pyogenes, which in turn is known to be a major virulence determinant involved in resistance to phagocytosis [39]. Within this viewer, expression based custom filters as well as custom queries based on calculated columns have been integrated for the quantification data for a set of predefined sample treatment conditions. In this example the S. pyogenes protein identification and quantification data have been adjusted to the biological question of interest. The quantification data have been aggregated on the treatment condition and the mean was calculated thereof. Additionally, the protein identifications have been filtered for proteins that have been induced upon plasma treatment (ratio of plasma treated and control samples > 5). Through the spreadsheet export functionality the quantification data of two UDP-glucose associated enzymes are finally downloaded and represented as a column plot (Figure 8C).

## Discussion

We have developed the open source software framework openBIS. This framework is easily extensible to store and process information from any biological experiment, it can flexibly handle metadata and is scalable to very large data. We have presented two of these extensions in this study, openBIS for Next Generation Sequencing and for Proteomics. We have created other versions of openBIS, specialized on other experiment types. Notably, we have built a version for HCS images, libraries and result data and a version for High-Throughput Metabolomics. We are currently working on integrating microscopy data, with a focus on light microscopy images.

There are several advantages of building extensible and customized systems on a core framework, both for the developers and the users. For the developers, building on a core framework provides a set of commonly required core functionalities "out of the box" and a template of how to add new functionality consistently and with low effort as they use well-defined extension points. From the user's perspective, in particular the data analysts and mathematical modellers who want to get data out of the system automatically or semi-automatically for down-stream analysis, access to data should be possible via custom APIs like e.g. a Matlab-API. Core functionalities, such as the browsing and searching of data, the management of metadata or permissions, the import and export of data are handled all in a consistent and familiar way. The ease of use of the software allows the researcher to focus on the data instead of becoming acquainted with a new application again.

In our view the extensibility of the framework using loose-coupling techniques is key to be able to adapt it to the various use cases and experimental technologies quickly since many of the required technologies regarding data ingestion, integration and analysis have yet to be developed. While data acquisition, processing and particularly analysis pipelines often change rapidly to incorporate the latest improvements in method development, data and information systems are expected to provide long-term stability. Thus, in our view a tight-coupling approach of data analysis and information systems is counter-productive.

The framework by itself with minor configuration work is useful for data and metadata management and provenance tracking. It answers questions like "What datasets has a given collaborator measured two years ago on the Orbitrap using HEK293 cells and what database searches did we do on it?" The effort for setting up a system for this purpose is less than 10% of the work to implement it from scratch and will work more reliable and scale better. For further data analysis, a bioinformatician traditionally will copy the datasets he is interested in to his laptop, write a script to query the relevant information from it, create a new representation and run all of it on the command line. In contrast, when investing the same work to write a reporting script for openBIS, he will be able to use the result to feed analysis programs, get an AJAX web page with the query result as a searchable table that can be used by biologists and will have it available as a web service call that can be used e.g. in a custom web page. The copy of the data on his laptop becomes optional.

Support for sophisticated research data workflows requires integration with analysis software and requires additional work to extend the system. As openBIS is well documented, it enables other laboratories to extend it themselves for their own needs [40]. Extensions within a domain, like adding new metadata models or writing a parser for a new measurement device, can be done by a biologist. Adapting the framework to a new biological domain, however, will require the know-how of a professional software engineer. The effort for creating such domain-specific extensions is significantly reduced, compared to writing the same functionality from scratch. Based on our experience, the effort is less than half.

It is becoming common practice to store all data with associated metadata, especially in data intensive disciplines. This is particularly important for large collaborative and distributed projects. The openBIS framework includes a flexible annotation service to store and search for a custom set of properties for experiments, samples and datasets by using property types. Any kind of custom sample submission form can be created. Alternatively, metadata can be imported from Excel sheets or automatically extracted from log files by the ETL process upon upload. Once stored, metadata can be used by the system to create custom export formats like the SOFT format required by NCBI GEO [41].

As high-throughput experimental approaches and usage of multiple measurement technologies within one project and distributed projects become prevalent in modern biology, scalability of biological information systems becomes an important issue. The framework we built is scalable for four reasons. First, the hybrid data store separates metadata and index data from bulk data, keeping the core data small enough to be fast to search. Second, queries in the core database are optimzed for performance. Third, multiple (possibly distributed) data stores are supported as part of one system, and each data store can use multiple data shares transparently to allow building very large repositories in a cost-effective way, and fourth, archiving datasets to cheap offline and near-online storage and bringing archived datasets back to online storage is an integrated feature of the framework.

openBIS for Next Generation Sequencing was developed for use in the Quantitative Genomics Facility of the Department of Biosystems Science and Engineering of ETH Zurich to support the organization, annotation and distribution of genomics data. To make the facility more efficient and high-throughput capable, openBIS is retrieving the data directly from the sequencer without raw images. The SRF files and quality metrics are generated after the sequencing ended and before the data will be put into openBIS. During the registration of a complete flow cell openBIS links the data to the already available metadata. Additional annotations to experiments, samples and datasets have been added as needed by defining new property types. Export and exchange of data and metadata for collaborators is additionally supported by CIFEX [30]. We are currently working on integration of openBIS with the Galaxy workflow system [15] that our data analysts like to use for further analysis of the sequencing data, as well as the UCSC Genome Browser [36].

openBIS for proteomics has been fully integrated into a proteomics workflow at the Institute of Molecular Systems Biology at the ETH Zurich. Raw data and metadata are captured from the mass spectrometers and stored in the openBIS database. P-Grade, a web portal framework with an integrated workflow management system has been integrated via an API with openBIS to support the building and execution of proteomics computational applications, such as TPP [42] or OpenMS [20]. Data resulting from processing pipelines are automatically imported into openBIS and linked to its raw data. Although the current system has been designed to handle protein identification and quantification data, it can also work with protein-protein interaction data, though we are aware of some possible improvements required for this use case. The integrated setup has many advantages. Researchers can perform mass spectrometric analyses much faster and with less manual steps. Based on the metadata stored in openBIS, the scientist can process the data externally and re-import them into openBIS. Search results become eventually available in openBIS and can be made accessible to collaborators easily. Due to the metadata stored in openBIS data can be tracked and handed over to researchers following up a particular mass spectrometric study. Furthermore, openBIS stored data and metadata can provide the basis for subsequent data integration into public resources such as the PeptideAtlas [43].

Today, it is possible to keep data from different measurement technologies on one openBIS server and to link these results based on biological samples, genes and proteins. However, framework support for this is limited. As more complex linking of result data from systems biology approaches is becoming standard, we believe that it will be a large area of further development to support this in the openBIS framework.

## Conclusions

openBIS provides the life sciences community with a useful software framework which is under active, continuous development. Multiple laboratories involved in several SystemsX.ch projects, EU-funded projects as well as institutional core facilities actively use it. Besides being a tool for data provenance tracking and a database for integrating with processing and analysis workflows and visualization tools, it is also used to publish data to collaborators and the scientific community, either directly or via reporting databases. While the focus of openBIS is to satisfy the needs of large-scale data handling platforms, it has been used successfully by smaller laboratories as well.

### Availability and requirements

• **Project name: **openBIS

• **Demo instance: **http://openbis-demo.ethz.ch

• **Project home page: **http://www.cisd.ethz.ch/software/openBIS

• **Documentation and download: **https://wiki-bsse.ethz.ch/display/bis

• **Operating system: **Mostly platform independent, some advanced functionalities require a modern Unix-like operating system like e.g. Linux

• **Programming language: **Java, Jython

• **Installation requirements**: Java Runtime Environment 1.6, PostgreSQL 9.0 or 9.1

• **License: **Apache Software License 2.0 (some libraries used in openBIS are available under other OSI-approved licenses)

• **Restrictions: **Access to helpdesk support and services requires an agreement with the Center for Information Sciences and Databases of ETH Zurich. Non-profit research organizations can get basic support for free.

## Authors' contributions

AB wrote a major part of the paper, tested and created user documentation of the software, IA, PB, FJE, KE, PG and TP wrote software, CR wrote software and contributed to the manuscript, MK contributed to the manuscript and integrated the software in a Next Generation Sequencing data pipeline and implemented extensions for this area, AQ drove bioinformatics requirements for proteomics data, tested the extensions for proteomics data and provided feedback, CB contributed the section on ChiP-seq data to the paper and co-designed the extensions for NGS, LM designed the extensions for proteomics data the integration in proteomics data workflows, RA contributed to the structure of the paper, and provided guidance and logistical support for the project, and BR designed the software, supervised the project and wrote part of the paper. All authors read and approved the final manuscript.

## Acknowledgements and funding

We thank Johan Malmström and Peter Kunszt for critical reading of the manuscript and Peter Kunszt and the SyBIT project of SystemsX.ch for creating a unique collaborative environment where openBIS could be developed. The authors also thank Adrian Honegger, Charles Ramin-Wright and Christian Ribeaud who have contributed to the project in the early development stage, and Claus Hultschig and Sean Walsh for contributing to requirements analysis in collaborative projects.

This project was funded by SystemsX.ch, the Swiss initiative for systems biology, the ETH Zurich, 3-V Biosciences, Inc. and by the 6th Framework Program of the European Commission through the AGRON-OMICS and BaSysBio Collaborative Projects.

## Supplementary Material

Additional file 1 **Glossary**. Glossary of terms used specifically in openBIS. Click here for file

Additional file 2 **Application and Data Store Server Architecture**. Both the AS and the DSS consist of three layers, the presentation layer, the domain layer and the data layer. Click here for file

Additional file 3 **Selected openBIS functionalities**. Core functionalities of openBIS as well as HCS, metabolomics, proteomics and next generation sequencing specific functionalities are listed in a table. Click here for file
