# Supplementary figures and images for: openBIS: a flexible framework for managing and analyzing complex data in biology research
Source: BMC Bioinformatics. 2011 Dec 8;12:468. doi: 10.1186/1471-2105-12-468 (PMC3275639; doi:10.1186/1471-2105-12-468)

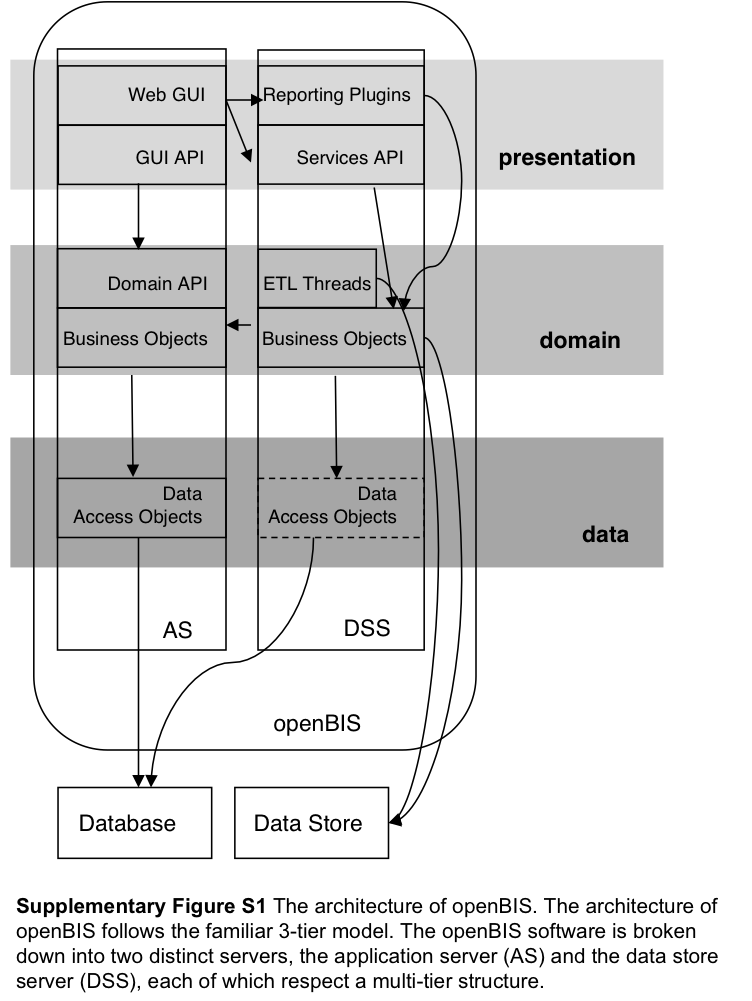

Supplement: Additional file 2 — Application and Data Store Server Architecture. Both the AS and the DSS consist of three layers, the presentation layer, the domain layer and the data layer. [file 1471-2105-12-468-S2.PNG]
